# Medical communication and technology: a video-based process study of the use of decision aids in primary care consultations

**DOI:** 10.1186/1472-6947-7-2

**Published:** 2007-01-10

**Authors:** Eileen Kaner, Ben Heaven, Tim Rapley, Madeleine Murtagh, Ruth Graham, Richard Thomson, Carl May

**Affiliations:** 1 Institute of Health and Society, The Medical School, Newcastle University, NE2 4HH, UK

## Abstract

**Background:**

Much of the research on decision-making in health care has focused on consultation outcomes. Less is known about the process by which clinicians and patients come to a treatment decision. This study aimed to quantitatively describe the behaviour shown by doctors and patients during primary care consultations when three types of decision aids were used to promote treatment decision-making in a randomised controlled trial.

**Methods:**

A video-based study set in an efficacy trial which compared the use of paper-based guidelines (control) with two forms of computer-based decision aids (implicit and explicit versions of DARTS II). Treatment decision concerned warfarin anti-coagulation to reduce the risk of stroke in older patients with atrial fibrillation. Twenty nine consultations were video-recorded. A ten-minute 'slice' of the consultation was sampled for detailed content analysis using existing interaction analysis protocols for verbal behaviour and ethological techniques for non-verbal behaviour.

**Results:**

Median consultation times (quartiles) differed significantly depending on the technology used. Paper-based guidelines took 21 (19–26) minutes to work through compared to 31 (16–41) minutes for the implicit tool; and 44 (39–55) minutes for the explicit tool. In the ten minutes immediately preceding the decision point, GPs dominated the conversation, accounting for 64% (58–66%) of all utterances and this trend was similar across all three arms of the trial. Information-giving was the most frequent activity for both GPs and patients, although GPs did this at twice the rate compared to patients and at higher rates in consultations involving computerised decision aids. GPs' language was highly technically focused and just 7% of their conversation was socio-emotional in content; this was half the socio-emotional content shown by patients (15%). However, frequent head nodding and a close mirroring in the direction of eye-gaze suggested that both parties were active participants in the conversation

**Conclusion:**

Irrespective of the arm of the trial, both patients' and GPs' behaviour showed that they were reciprocally engaged in these consultations. However, even in consultations aimed at promoting shared decision-making, GPs' were verbally dominant, and they worked primarily as information providers for patients. In addition, computer-based decision aids significantly prolonged the consultations, particularly the later phases. These data suggest that decision aids may not lead to more 'sharing' in treatment decision-making and that, in their current form, they may take too long to negotiate for use in routine primary care.

## Background

Clinicians increasingly recognise the importance of engaging patients in health care especially where decisions about treatment have significant effects on quality of life or where there are choices between alternative therapies with differing risks and benefits [1,2]. Indeed for ethical reasons, patients' autonomous authorization of medical interventions (informed consent) is usually required before treatment can or should occur [3]. In primary care, increased patient engagement in health care is reflected in a range of interventions aimed at developing patient-centred approaches during consultations which take into account patients' needs and anxieties concerning treatment [4,5]. Such approaches sit well with the acknowledgement that diagnostic uncertainty is common for primary care clinicians [6,7] and that patients are experts regarding the experience of their health condition with legitimate preferences for differing health states, treatments and outcomes [8].

Until recently, research on patient-centred approaches tended to focus on promoting a joint understanding of patients' problems which generally occurs earlier in the diagnostic stages of a consultation [9]. Less emphasis was placed on treatment decision-making which tends to occur later in the consultation. However, the requirement for informed consent for medical treatment [3], coupled with the fact that increasing numbers of patients expect to be involved in decisions about their care [10], has led to the development of numerous patient decision aids aimed at promoting shared decision-making. Shared decision-making has been defined as a collaborative endeavour in which:

'both patient and doctor have a legitimate investment in the treatment decision; hence both declare treatment preferences and their rationale while trying to build consensus on the appropriate treatment to apply' [11]

There has been a prolific expansion in patient decision aids aiming to promote shared decision-making over the past 10 years which has been aided by recent technological advancements in health care, and so patient decision aids now take on a wide range of forms from written pamphlets to interactive computer programmes [12,13]. Most of the evaluative work on decision aids has looked at consultation outcomes and found that they can improve patients' knowledge about a condition and reduce decision conflict about treatment; although they have had a variable impact on the actual decisions made and on satisfaction with treatment [14]. However, treatment decision-making is complex and dynamic and there can be many shifts and changes in clinicians' and patients' style of involvement in a discussion and/or use of a decision aid as a consultation proceeds [15]. Relatively little is known about the impact of decision aids on the format, trajectory and style of consultations. The ease, or conversely unease, with which technologies like decision aids are embedded in practice is important since patients' perceptions about the quality of health care communication within consultations can affect their health and even their prognostic outcomes after treatments [16,17].

Thus we need to understand how decision aids are actually used by clinicians and patients in health care [10] including their impact on the content and timing of the consultation. Given the variety of forms that decision aids can take, it is also essential to assess the relative impact that different technologies might have on the course of the clinical encounter [18].

The aim of this study was to quantitatively describe the impact of three types of decision aid on the duration and content of consultations focused on treatment decision-making in primary care. This quantitative observational work was part of a larger mixed-method (video and interview-based) process study of a randomised controlled trial. The parent trial aimed to promote decision making in primary care via the use of three arms: two computerised patient decision aids delivered in a shared decision making consultation; and a control condition of paper-based guidelines applied in a paternalistic way as a source of doctor-led advice. In the process study, we did not begin with a specific hypothesis concerning the impact of these decision aids on the consultation. In line with the qualitative research tradition, we wished to inductively identify and describe the differences in interactional behaviour between the three arms. However, this involved two informal hypotheses. (i) that differences in the three arms of the trial would lead to differently structured interactions between participants, and (ii) that the use of technology in the decision aid arms would affect the inter-personal communication.

The observational component of the process study reported in this paper involved a detailed interaction analysis of consultation behaviour using numeric data to facilitate comparison between three different arms of the trial. This observational work was informed by the precepts of ethology: non-participant observation of subjects in a naturalistic context accompanied by systematic data sampling and analysis of manifest behaviour [19]. This naturalistic approach to the study of behaviour avoids imposing a priori conceptualizations on the target activity and is compatible with grounded theory approaches to research [20,21].

## Methods

### Trial context

The parent trial was an efficacy study of the use of the computerised patient decision aids [22] in primary care consultations. The patient decision aids were designed to support decision-making concerning warfarin anticoagulation or aspirin treatment to reduce the risk of stroke in patients with atrial fibrillation [23,22]. There were two versions: the implicit (concise) patient decision aid involves individualised risk and benefit presentation and a section to support shared decision-making; the explicit (extended) patient decision aid additionally includes patients' elicited values for health and treatment states derived via standard gamble and analysed in a Markov decision analysis [23]. This latter version therefore incorporated not only personalised risk and benefit data, but also derived personal values (utilities) for the relevant health states which were used in a decision analysis. The output of the decision analysis was presented to the patient to support the shared decision making section of the consultation.

The three armed trial sought to determine whether the two versions of the computerised decision aid, applied in the context of shared decision-making, were efficacious at reducing decisional conflict compared to a control condition of paper-based clinical guidelines derived from the same decision analysis and applied as a doctor-led source of advice. In each arm of the trial, a single male GP administered one version of the decision aid. These three GPs were familiar with their version of decision aid and trained to deliver it in as close to optimum conditions as possible. Patients were randomly allocated to the three arms of the trial and experienced only one form of decision aid. Consultations in this trial acted as referral clinics; any decisions made were forwarded to the participants' own GP who retained overall clinical responsibility. The trial GPs were therefore not the patients' usual GP, nor were clinics held in their usual surgeries.

### The process study

The process study was carried out between January 2003 and April 2004 on 29 out of the first 31 trial consultations. There were 10 patients in the guidelines arm, 11 patients in the implicit tool arm and 8 patients in the explicit tool arm. We anticipated more subjects in the explicit tool arm, however observed difficulties for patients in understanding the standard gamble procedure subsequently led to the explicit arm of the trial being discontinued prematurely [24].

Ethical approval for both the trial and the process study was granted by the Newcastle and North Tyneside Local Research Ethic Committee and research governance approval by North Tyneside Primary Care Trust.

The process study was in three inter-linked parts:

(a) video-recording of consultations followed by a quantitative interaction analysis

(b) conversation analysis of consultations

(c) in-depth qualitative interview work with patients, 3 days and then three months after the consultation.

The detailed methods and results of (b) and (c) are reported elsewhere [25,26] and this paper focuses on the quantitative observational work (a).

The videos enabled non-participant observation of subjects during the consultations. Twenty nine consultations were video-recorded using an unmanned digital camera, positioned so that the upper bodies of participants and the decision-support tools were visible. Video-recordings of the consultations were imported into the Observer software to facilitate coding of verbal and non-verbal behaviour and later interaction analysis [27]. The audio-stream of data was also fully transcribed to facilitate data coding.

### Methodology

Interaction analysis is a systematic analysis of the clinical encounter via identification, categorization and quantification of salient features of doctor-patient communication [28]. The coding-frame for verbal behaviour was based on the Medical Interaction Process System [MIPS] [29], and the Roter Interaction Analysis System [RIAS] [30], both well established protocols for clinical communication research [31]. Utterances, defined as the smallest classifiable segment of speech [29,31], were the coding unit for verbal behaviour. Utterances were coded by mode of behaviour (e.g. open or closed questioning) and, where appropriate, by content (e.g. medical, psychological or social focus). Given the importance of non-verbal behaviour in communication [32], particularly in supporting verbal behaviour [33], non-verbal codes were added to the coding frame. These were initially derived from observing six simulated GP-patient consultations, developed for training purposes, and cross-checked against published work in this field [34-36].

The final version of the coding frame is appended [see Additional file 1] and an explanatory manual is available on request. Codes included 23 verbal modes of behaviour (8 with content classification) which were coded as they occurred. There were also 9 non-verbal activities which were coded. Rapid activities (e.g. smiling or nodding) were coded as they occurred whilst slower behaviour was discontinuously coded at 1 minute sample intervals (Martin & Bateson 1986). Slow behaviour which could change suddenly (e.g. gaze) was coded using 'one-zero sampling' in which any changes within the previous minute were recorded. Slow and prolonged behaviour (e.g. posture) was coded using 'instantaneous sampling' in which only the behaviour occurring at the minute time-point was recorded. Nonverbal behaviour involving touching or pointing was classified as to whether it was directed at the other person or the decision aid.

### Data analysis

Analysis aimed to combine a broad overview of the consultation (molar approach) with a detailed description of a sampled section (molecular approach) of the consultation as recommended for communication research [31]. The overall duration of consultations was measured, as was the duration of different phases within it [37]. These phases included: opening activity (greeting and explanatory work); physical examination; presentation of risk information; decision-making discussions; and closure work. In addition, the duration of standard gamble procedures was recorded in explicit decision aid consultations.

Due to the high volume of complex data available in videos, a section of the consultation was sampled for detailed study. Henbest [38] found that scoring a section of consultations can be as reliable as using the entire consultation. Moreover, a study of different sample-lengths found that a 10-minute 'slice' was a reliable proxy for the entire consultation for verbal and non-verbal behaviour [34,39]. Since the focus of the trial was decision-making, a ten-minute 'slice' of activity leading up to the decision-point was sampled for detailed analysis.

Verbal behaviour was collated into established meta-groupings [40,41] [see Additional file 2] as recommended for this type of research [42]. These meta-grouping make intuitive sense of the behaviour, reduce the risk of multiple comparison testing and enable comparison across other studies in this field.

Finally, a measure of verbal dominance was calculated as a percentage of all GP utterances divided by total GP + patient utterances.

### Reliability of coding

The reliability of data coding was established by intra and inter-observer reliability statistics. Pearson's r correlations at the 1% significance level for both reliability measures were calculated for all behaviour categories with mean frequency greater than 2. One researcher (BH) initially coded all 29 consultations. For intra-observer reliability, the same researcher recoded a one-minute random sample of activity from the 29 consultations six-months after initial coding. The overall average intra-observer correlation was 0.87; verbal behaviour had a mean correlation of 0.90 (range 0.87–0.93) and non-verbal behaviour had a mean correlation of 0.83 (range 0.82–0.84). For inter-observer reliability, a second researcher (TR) coded a one-minute sample from the initial 29 'slices' of the consultation. The overall average inter-observer correlation was 0.80; verbal behaviour had a mean correlation of 0.81 (range 0.79–0.82) and non-verbal behaviour had a mean correlation of 0.80 (range 0.72–0.85).

### Statistical issues

The analysis was intended to be primarily descriptive, although statistical tests were used to enable comparison across the three trial conditions (types of consultation). Since the number of consultations was relatively small (n = 29) and because the data were not normally distributed, non-parametric statistics were used. Thus median summary values plus quartiles were reported. Statistical significance was set according to the convention level of P < 0.05.

## Results

The median age of the 29 patients in the study was 72 years (quartiles 67–77), of which 13 were women (median age 73, quartiles 69–79) and 16 were men (median age 69, quartiles 66–73). There were no significant differences in age or sex in the 3 trial groups. Most patients (n = 24, 83%) entered the trial on warfarin medication, three were taking aspirin and two were not on treatment prior to the trial.

### Consultation timing

**Figure 1:**
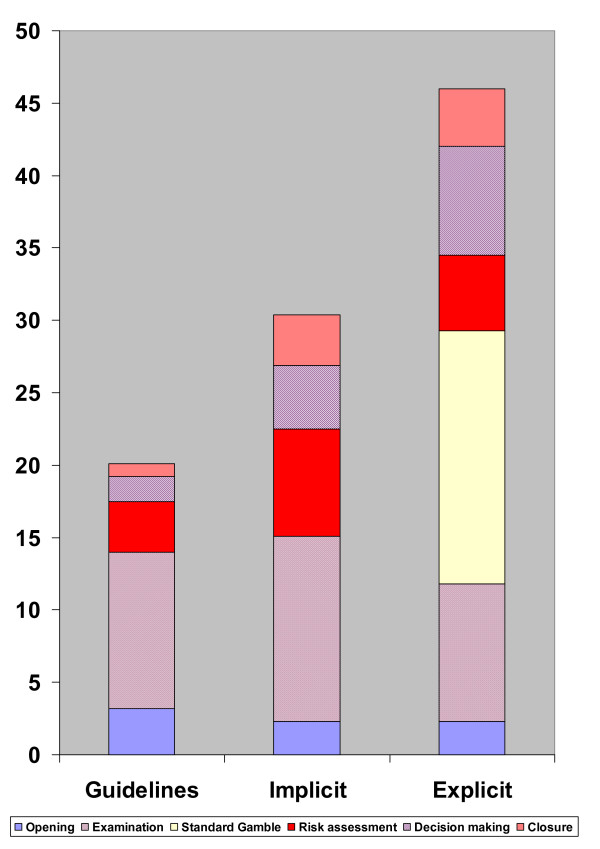
Median duration of the different phases of the consultation shown in minutes.

Across all 29 consultations, the median consultation time was 29 minutes (quartiles 20–43 minutes). There was a significant difference in consultation times across the three arms of the trial (Kruskal Wallis χ^2 ^= 13.8, df 2, P = 0.001). The median consultation times were: 21 minutes in the guidelines consultations (quartiles 19–26 minutes); 31 minutes in implicit tool consultations (quartiles 16–41 minutes); and 44 minutes in explicit tool consultations (quartiles 39–55 minutes). Within these consultations, there was a significant difference in timing of the phases focused on risk assessment (Kruskal Wallis χ^2 ^= 16.2, df 2, P < 0.001), decision-making (Kruskal Wallis χ^2 ^= 8.3, df 2, P = 0.014) and closure (Kruskal Wallis χ^2 ^= 16.2, df 2, P < 0.001). These three later phases were shortest in consultations involving paper-based guidelines as shown in Figure 1.

Table 1 shows the timings for individual patients in the order that consultations occurred in the trial. Despite the fact that one GP delivered each arm of the trial, there was considerable variability both within and across the arms of the trial. Thus each GP had longer and shorter consultations. However, consultations involving computerised decision aids were generally longer than those involving paper-based guidelines. In addition, there were no obvious GP learning effects over time; that is, consultations did not get shorter as a GP became more familiar with the technology. It was clear that the standard-gamble procedure of the explicit tool dominated the middle section of consultations and extended their overall timescale. In these 8 consultations, the standard gamble exercise took up over a third of the entire consultation time (modal value 35%) with a range of 28% to 51%.

### Consultation content

In the 10 minutes immediately preceding the decision point, GPs dominated the conversation with an overall median verbal dominance score 64% (quartiles 58–66%). GPs' verbal dominance across the trial arms was 60% (quartiles 55–64) in the guidelines consultations; 65% (quartiles 63–69) in the implicit tool consultations; and 64% (quartiles 60–66) in the explicit tool consultations. These differences in verbal dominance across the trial arms were not statistically significant (Kruskal Wallis χ^2 ^= 4.7, df = 2, P = 0.09). Most of the GPs' conversation was technical (93%) rather than socio-emotional (7%). For patients, the respective proportions were 85% and 15%. The tendency to focus on technical rather than socio-emotional matters did not significantly differ across the three arms of the trial, for GPs or patients.

The frequencies of different modes of verbal behaviour shown by GPs and patients are presented in Table 2. Information-giving was the dominant activity for both GPs and patients, followed by paralinguistic registering of the other persons' talk (e.g. uh huh). There was a significant difference by trial arm in the frequency of information-seeking by GPs which occurred least in implicit tool consultations and in their conversational pauses which were shown most in the guidelines consultations. For patients, there was a significant difference by trial arm only in negative talk which occurred least in the implicit tool consultations.

Frequencies of non-verbal behaviour shown by GPs and patients are shown in Table 3. Head nodding, a common way of indicating attention in conversation, was most frequent activity for GPs and patients. For GPs, there was a significant difference by trial arm in several non-verbal activities: nodding, head-shaking, smiling, pointing at the patient, touching or pointing at the decision aid and eye-gaze directed towards the decision aid. In the guidelines consultations, the GP showed the least nodding, smiling and tool-directed eye-gaze but the most head-shaking and pointing to patients. For patients, only tool-directed eye-gaze differed by trial arm and this occurred least in guidelines consultations.

## Discussion

All the consultations in this study took considerably longer than the time usually available in primary care, approximately ten minutes [43]. Thus it is unlikely that, in their current form, the paper-based or computerised decision aids can be easily incorporated in GPs' routine consultations. Centrally, both computerized versions of the DARTS II tool prolonged the risk assessment and decision making phases compared to paper-based guidelines. These computerised decision aids were applied in the context of shared-decision making and it has been noted that actively involving patients in treatment decisions requires additional time [44,45]. The computerised decision aids also prolonged the closing phases of the consultation. Reviewing the videos revealed that the GPs were often engaged either in procedural work such as printing off summary reports from the tool or in explaining the 'role' of the computer in the consultation.

In the 10 minutes leading up to the decision point, information-giving and technically-focused conversation dominated proceedings. Although patients' talk was primarily technical, it contained twice the socio-emotional content of the GPs' conversation. Given that the socio-emotional or affective component of clinicians' talk is a key factor in patient's positive evaluation of consultations [46] and subsequent adherence to treatment [47], it is essential to establish if the use of technology encourages a shift towards technical language at the expense of inter-personal work. Our results showed no significant difference across the arms of the trial in the proportion of technical to socio-emotional talk shown by GPs. Only the length of time spent working through the tools differed. Thus the technological complexity of the decision aid did not seem to affect the balance of technical to socio-emotional language used by GPs. However, future work should compare the impact of decision aids (in any form) on the technical/affective language balance compared to general consultation discourse.

What was consistent across all consultations in this study was the large amount of information provided to patients which tended to increase as the sophistication of the decision aids increased, albeit with a time cost. Overall, the GPs in this study spent 55% of the consultation giving information and 7% seeking it, which differs from the average proportions of 35% and 23% reported in general medical dialogue [41]. The patient proportions of 33% and 7% in this study compare with 54% and 6% reported elsewhere [41]. In this respect the decision aids appeared to be useful tools in presenting patients with information, suggesting that they might encourage an 'informed' model of treatment decision-making [48] where the clinician provides all the relevant information to patients who then select the treatment they deem most appropriate [5]. However, the enactment of 'shared' decision-making may remain dependant on the GP/patient social interaction and not directly shaped by technological decision aids.

It was clear that GPs in this study conversationally led the consultation, contributing nearly two-thirds of all utterances and showing more pauses within their speech than patients. More powerful conversants generally talk for longer and are allowed more uninterrupted pauses in their speech [49]. The verbal dominance seen in this study is similar to the average 60:40 ratio (doctor:patient) reported in general medical dialogue [41]. Indeed we observed no significant difference in verbal dominance, or clear differences in verbal content, between the 'shared decision making' consultations of the computerised decision aids and the 'paternalistic' consultations involving paper-guidelines. This may appear counter-intuitive, in that one might expect shared decision making consultations to have less verbal dominance than paternalistic consultations. However, it may be that it takes considerable verbal work from doctors to introduce and sustain any model. In addition, such models do not just occur and an initial intention to share may get lost in the unfolding dynamic of the consultation [49], particularly where clinicians are tasked to work with and through a 'third party' in the consultation, in the form of a decision aid.

GPs and patients both seemed behaviourally engaged in these consultations, showing a great deal of paralinguistic registering of the conversation [34]. There were a number of significant differences in GP's nonverbal activity across the arms of the trial but no consistent trends. Nevertheless, nonverbal activity appeared to be a key aspect of communication and we found a great deal of mirroring in eye-gaze and illustrative gesturing [49]. For example, on one occasion a GP was attempting to explain atrial fibrillation to a patient and he used his hands to demonstrate the pumping and fluttering action of the heart. Thus it is clear that unique information can be conveyed visually [50]. Indeed it has been reported that just 7% of emotional communication is conveyed verbally compared to 22% via voice tone and 55% through visual cues [32]. However, although non-verbal behaviour has long been regarded to be an important feature of good communication [51] and an influence on medical outcomes such as patient understanding, compliance and satisfaction with care [52] it is much less well understood than verbal behaviour [53]. Thus more attention should be focused on identifying and understanding the contribution of non-verbal behaviour to communication in clinical contexts.

### Data limitations

Due to the exploratory nature of the process study, particularly the fact that each video-taped consultation was linked to two subsequent in-depth interviews which was highly labour intensive, the number of patients in this observational analysis was relatively small. Moreover, since the data were not normally distributed, we used conservative non-parametric statistics in our analysis, which was intended to be primarily descriptive. Thus it is possible that real but small differences between the three types of consultation were not detected which may have emerged with a larger sample. Also the efficacy design of the trial meant that just one GP delivered each decision aid and so our findings may be confounded by GPs' style in consultations rather than the decision aid per se. Nevertheless, the GPs' verbal dominance and technical focus was apparent across all the consultations on this study.

Although patients were randomised into the parent trial, we used a convenience sample of the first 29 patients who agreed to be videotaped (31 were asked). The trial eventually recruited 109 patients [54]. In addition, the earlier recruits in our study are more likely to be prevalent cases in the population with a longer experience of atrial fibrillation compared to later incident cases. Moreover, the third arm of the trial (explicit version of DARTS II) was discontinued after eight consultations as a result of data produced by process study. We observed that a number of patients found it difficult to understand the preference elicitation exercise in these consultations and were uncomfortable with this activity [24]. Thus this divergence between the early process study and the final trial clearly limits our ability to generalise findings from the former to the latter. However, the relative lack of differences in patients' behaviour across the differing types of consultations may help explain why the trial found only transient effects of a computerised decision aid on patients' decision conflict and no difference in actual treatment decisions [54].

Due to the complexity of video-based data, a 10 minute slice was sampled for detailed content analysis. The fact that GPs in this study were so focused on information-giving may reflect the fact that conversational content varies in different phases of a consultation [37] and this work focused on the time just before the decision point. However, within the consultations promoting shared decision-making this should have been the time when patients were most actively involved in the conversation. We found that they were much less vocal than the GPs at this crucial time point.

In addition, consultation timing varied depending on which decision aid was used and so the sampled 'slice' of the consultation may have crossed differing sub-phases. For instance, some physical examination work was included in most time-samples from guidelines consultations, some from implicit tool consultations and none from the explicit tool consultations. However, the lack of clear differences in verbal behaviour between the trial arms suggests that the latter did not strongly affect our analysis.

Perhaps most importantly, the patients in this study were also participants in a trial who had experienced extensive consent procedures, who were not seeing their own GP and who were attending an unfamiliar clinic. This context may further explain the lack of differences in treatment decisions reported by the trial [54]. Subsequent interview work indicated that some participants regarded themselves as 'subjects' in a research study rather than 'real' patients [26]. Moreover, conversation analysis of these consultations revealed how the experimental context of these consultations, at moments, overwhelmed the clinical context of the interaction [25]. In this way, the impact of the trial context on both patients and clinicians may have outweighed any impact of the decision aids themselves. For patients, the eventual treatment decision may not have been perceived as 'real' and for clinicians the unfamiliar patients may have prompted less socio-emotional language than usual and more conversational work to maintain the dialogue.

Although the efficacy trial design (with high internal validity) may limit the applicability of our process data to the 'real world' of primary care, it is important to evaluate the impact and acceptability of complex interventions, such a decision aids, on a limited sample of patients (and indeed clinicians) before wider scale roll out. The work in this study revealed significant difficulties for patients with the extended version of the DARTS II tool, despite earlier pilot work. We also found that working with this decision aid took clinicians and patients much longer than the time usually available for consultations in primary care. Thus further development of this decision aid may be required before the next evaluative stage which should incorporate a more pragmatic (clinically representative) study design.

## Conclusion

This study showed clear differences in the duration of primary care consultations depending on the form of decision aid used. The use of paper-based guidelines took twice the time usually available in primary care and computerised decision aids took three or four times longer than a standard consultation. Thus, unless specific clinics are set aside for such work, it is unlikely that traditional or technologically focused decision aids will be easily incorporated into routine primary care. Furthermore, even in consultations aimed at promoting shared decision-making, GPs were verbally dominant and more technically focused than patients. Moreover despite differences in the technological sophistication of the decision aids, we found almost no difference in behaviour indicative of shared decision-making across the trial arms. Responsibility for the enactment of a shared decision-making consultation may therefore remain within the interactional space between GP and patient. Decision aids may well be closer to their designation than currently perceived; optional resources which foster information transfer rather than shared decision-making machines. Our data showed the extensive use of these tools in presenting complex information to the patient. However, we found no clear evidence that they promoted the sharing of treatment decisions.

## Competing interests

The author(s) declare that they have no competing interests.

## Authors' contributions

EK, CM and RT conceived the original design for the process study and secured funding for the study.

BH and TR carried out the data collection for the observational part of the process study. BH, MM and RG carried out the data collection for the linked qualitative interview work.

EK carried out the main data analysis; BH and TR carried out inter and intra-observer reliability analyses. EK drafted the paper and BH wrote part of the method section.

All authors provided comments on the manuscript and helped with its redrafting.

## Pre-publication history

The pre-publication history for this paper can be accessed here:



## Supplementary Material

Additional file 1 Appendix 1. Coding Frame. The coding frame used to code observational data. Click here for file

Additional file 2 Appendix 2. Meta-groupings of behaviour. The grouping of individual behaviour patterns into broader categories of activity. Click here for file

## Figures and Tables

**Table 1:** Timing of consultations and their phases in minutes

**Patient**	**Opening**	**Examination**	**Standard Gamble**	**Risk Assessment**	**Decision Making**	**Closure**	**Total**
*Guidelines*							
1	4.2	10.2	n/a	3.5	1.2	0.9	20.0
2	3.0	16.2	n/a	3.5	1.4	1.6	25.7
3	2.6	10.7	n/a	2.9	1.2	1.0	18.4
4	4.6	14.5	n/a	4.0	3.4	0.8	27.3
5	3.4	9.3	n/a	5.2	10.1	0.3	28.3
6	2.0	10.9	n/a	3.5	1.6	0.8	18.8
7	6.9	9.4	n/a	1.1	1.8	0.4	19.6
8	3.1	11.4	n/a	2.9	5.3	1.7	24.4
9	3.3	14.0	n/a	3.4	0.7	0.6	22.0
10	2.4	8.4	n/a	4.7	1.8	2.9	20.2
*Implicit*							
1	2.3	18.2	n/a	12.6	6.8	4.5	44.4
2	3.9	14.0	n/a	8.4	3.0	2.3	31.6
3	0.7	12.8	n/a	9.8	12.1	5.6	41.0
4	1.0	14.3	n/a	7.7	4.4	7.0	34.4
5	6.8	21.5	n/a	5.9	5.2	7.9	47.3
6	2.4	3.9	n/a	5.7	0.9	3.5	16.4
7	2.5	3.2	n/a	5.6	1.9	2.2	15.4
8	1.4	2.7	n/a	7.4	1.9	2.0	15.4
9	0.8	13.0	n/a	9.7	3.5	1.8	28.5
10	3.3	9.4	n/a	7.3	7.3	3.6	30.9
11	1.6	8.3	n/a	7.3	5.4	2.3	24.9
*Explicit*							
1	2.5	1.4	21.9	9.7	2.4	5.0	42.9
2	1.1	10.4	14.3	8.9	8.1	8.8	51.6
3	1.6	6.6	13.2	6.5	7.6	2.5	38.0
4	2.0	7.0	19.0	5.1	7.7	2.4	43.2
5	3.1	14.1	20.9	3.4	9.1	5.6	56.2
6	2.7	9.4	25.9	4.6	7.5	9.1	59.2
7	4.6	10.6	16.0	5.2	6.9	1.7	45.0
8	1.3	9.6	10.5	3.1	2.0	3.1	29.6

Footnote: cases in each condition are in the order they occurred in the trial

**Table 2:** Median frequency (quartiles) of verbal behaviour

	Guidelines n = 10	Implicit n = 11	Explicit n = 8
*Doctors*			
Information-giving	31 (27–39)	40 (37–57)	42 (31–47)
**Information-seeking**	**6 (5–10)**	**3 (3–5)**	**7 (6–9)**
Social talk	1 (0–1)	0 (0-0)	0 (0–1)
Positive talk	5 (3–9)	6 (2–10)	8 (6–9)
Negative talk	1 (0–1)	0 (0-0)	0 (0–1)
Partnership building	3 (3–4)	3 (1–5)	3 (2–5)
Registering other	18 (12–23)	9 (6–20)	20 (13–25)
**Pause**	**6 (3–7)**	**4 (1–6)**	**1 (0–4)**
			
*Patients*			
Information-giving	17 (12–21)	16 (7–21)	14 (12–21)
Information-seeking	3 (1–5)	3 (2–3)	5 (2–9)
Social talk	1 (0–2)	0 (0–1)	0 (0–2)
Positive talk	12 (11–15)	7 (5–18)	12 (10–13)
**Negative talk**	**2 (0–3)**	**0 (0–1)**	**1 (1–2)**
Partnership building	0 (0–1)	0 (0-0)	0 (0–1)
Registering other	15 (6–23)	8 (2–18)	11 (7–25)
Pause	1 (1–4)	1 (0–1)	0 (0–1)

Footnote: Items in bold indicate statistically significant differences across the arms of the trial using Kruskal Wallis tests. Doctors'behaviour: Information seeking χ^2 ^= 11.2, df 2, P < 0.004 and Pauses χ^2 ^= 6.1, df 2, P < 0.04. Patients' behaviour: negative talk χ^2 ^= 8.7, df 2, P < 0.01.

**Table 3:** Median frequency (quartiles) of non-verbal behaviour

	**Guidelines n = 10**	**Implicit n = 11**	**Explicit n = 8**
*Doctors*			
**Nodding**	**17 (15–20)**	**36 (29–45)**	**21 (18–28)**
**Head shake**	**4 (2–5)**	**2 (1–3)**	**0 (0–1)**
**Smiling**	**0 (0-0)**	**1 (0–3)**	**2 (0–3)**
Touching the patient	0 (0-0)	0 (0-0)	0 (0–1)
**Pointing at the patient**	**1 (0–2)**	**0 (0-0)**	**0 (0-0)**
**Touching/pointing at tool**	**6 (3–8)**	**1 (1–4)**	**6 (4–12)**
Hand gesturing (illustrative)	9 (4–15)	11 (4–21)	13 (4–15)
Eye-gaze towards patient	15 (12–18)	19 (16–20)	19 (17–20)
**Eye-gaze towards tool**	**5 (3–7)**	**15 (14–19)**	**16 (16–18)**
Position inclined to patient	1 (0–2)	0 (0–3)	0 (0–1)
Position inclined to tool	2 (0–4)	1 (0–2)	1 (0–2)
			
*Patients*			
Nodding	21 (16–34)	31 (13–39)	19 (15–31)
Head shake	3 (1–6)	3 (2–7)	4 (2–7)
Smiling	2 (1–3)	0 (0–1)	1 (0–2)
Touching the doctor	0 (0-0)	0 (0-0)	0 (0-0)
Pointing at the doctor	0 (0–1)	0 (0-0)	0 (0-0)
Touching/pointing at tool	0 (0–1)	1 (0–3)	0 (0–1)
Hand gesturing (illustrative)	3 (2–7)	5 (3–10)	6 (2–7)
Eye-gaze towards doctor	15 (12–18)	19 (17–20)	17 (16–18)
**Eye-gaze towards tool**	**5 (3–6)**	**16 (14–17)**	**16 (15–17)**
Position inclined to doctor	1 (0–2)	0 (0–3)	0 (0–1)
Position incline to tool	2 (0–4)	1 (0–2)	1 (0–2)

Footnote: Items in bold indicate statistically significant differences across the arms of the trial using Kruskal Wallis tests. Doctors' behaviour: nodding χ^2 ^= 10.5, df 2, p < 0.005; head shaking χ^2 ^= 10.1, df 2, P < 0.006; smiling χ^2 ^= 6.4, df 2, P = 0.04, pointing at the patient χ^2 ^= 8.4, df 2, P = 0.01 and touching/pointing at the decision aid χ^2 ^= 10.0, df 2, P = 0.007 and eye-gaze towards the decision aid χ^2 ^= 18.4, df 2, P < 0.001. Patients' behaviour : tool directed eye-gaze χ^2 ^= 17.3, df 2, P < 0.0001.
