# Supplementary material for: Medical communication and technology: a video-based process study of the use of decision aids in primary care consultations
Source: BMC Med Inform Decis Mak. 2007 Jan 10;7:2. doi: 10.1186/1472-6947-7-2 (PMC1781432; doi:10.1186/1472-6947-7-2)
Supplement: Additional file 1 — Appendix 1. Coding Frame. The coding frame used to code observational data. [file 1472-6947-7-2-S1.doc]

**Appendix 1 - Coding Frame**

**Verbal behaviour**

*Dependent* *mode (requires content categories)*

Asks open question [asks OQ]

Asks closed question [asks CQ]

Gives information [gives info]

Gives reassurance [gives reas]

Checks information/checks understanding [ch info]

Directs/advises [drcts/ads]

Summarises [sum]

Interrupts [intrpt]

*Independent mode (does not require content categories)*

Orientation/instruction [orient]

Shows agreement/understanding/positive response [agree]

Disagreement/negative response [dis]

Positive Exclamation [gives +E]

Negative Exclamation [gives –E]

Registers information [regs]

Empathy/Support [gives emp]

Laughs [lghs]

Asks for repetition [asks rep]

Expresses irritation [exp irrit]

Expresses gratitude [exp grat]

Expresses apology [exp apol]

Social conversation/personal remarks [social]

Unintelligible [unintell]

Pause [pause]

*Content Categories:* Medical condition [Med]; Other Medical [OMed]; Treatment [Treat]; Side-effects [side]; Psychological/medical [Psy/Med]; Psychological [Psy]; Lifestyle [life]; Social/Demographic [Soc/dem]; Administrative/practical [Ad/Prac].

**Non-verbal behaviour**

*Continuously recorded (as they occurred)*

Nodding

Head shaking

Smiling

Touch (self/other/tool/object)

Point (self/other/tool/object)

Hand gestures (illustrative or batonic)

*Discontinuously recorded (at 1 minute intervals)*

Gaze (in previous time interval)

Posture (on the time point)

Spatial Positioning (in previous time interval)
