# Supplementary material for: Medical communication and technology: a video-based process study of the use of decision aids in primary care consultations
Source: BMC Med Inform Decis Mak. 2007 Jan 10;7:2. doi: 10.1186/1472-6947-7-2 (PMC1781432; doi:10.1186/1472-6947-7-2)
Supplement: Additional file 2 — Appendix 2. Meta-groupings of behaviour. The grouping of individual behaviour patterns into broader categories of activity. [file 1472-6947-7-2-S2.doc]

**Appendix 2. Meta-groupings of behaviour**

**Mode**

Information-giving: Information-giving; directing/advising talk; summarising; orientation/instruction

Information seeking: Open or closed questioning, asking for repeated information

Social talk: Social conversation; personal remarks

Positive talk: Agreement, positive exclamation, reassurance; expression of gratitude; empathy, laughter/joking

Negative talk: Disagreement; negative exclamation; interruption; expression of irritation

Partnership building: Checks information or understanding

Pauses Breaks in speech made by the speaker

Registering Para-linguistic registering of the other person’s talk

**Content**

Technical: Content that covers medical, treatment, side-effects or administrative/practical matters about care

Socio-emotional: Content covering psychological, lifestyle or social issues
